# Genome-wide genotyping and SNP discovery by ultra-deep Restriction-Associated DNA (RAD) tag sequencing of pooled samples of *E. grandis* and *E. globulus*

**DOI:** 10.1186/1753-6561-5-S7-P45

**Published:** 2011-09-13

**Authors:** Dario Grattapaglia, Sergio de Alencar, Georgios Pappas

**Affiliations:** 1 EMBRAPA Genetic Resources and Biotechnology – Estação Parque Biológico, 70770-910 and Genomic Sciences Program - Universidade Catolica de Brazilia, 70790-160 Brazilia, DF, Brazil; 2 EMBRAPA Genetic Resources and Biotechnology – Estação Parque Biológico, 70770-910, Brazilia, DF, Brazil

## Background

The availability of next generation sequencing (NGS) technologies has opened the door to new strategies of SNP discovery and genotyping. Rapid genome-wide SNP detection via deep resequencing of reduced representation libraries of restriction digested pools of genomic DNA combined with a reference genome has been successfully used for SNP discovery in microorganisms [1], plants [2]and domestic animals [3]. Taking a step further from using NGS for SNP discovery, Baird et al [1]showed that NGS of short tags derived from barcoded multiplexed genomic representations generated with restriction enzymes could be used for direct genotyping of individuals, calling this method RAD (Restriction-site associated DNA) sequencing. RAD sequencing involves cutting a genome with at least one restriction enzyme and NGS the ends of the resulting fragments. We have recently developed a first set of SNPs for high-throughput genotyping of species of *Eucalyptus*. Although SNP assay success was high, the proportion of polymorphic SNPs declined as phylogenetic distance between species increased, down to <20% when contrasting *E. grandis* and *E. globulus*, the two main worldwide commercially planted species were considered [4]. In this work we used RAD sequencing to discover polymorphic SNPs across these two species. Additionally we were interested in assessing the potential of RAD for direct genotyping-by-sequencing in *Eucalyptus*.

## Methods

DNA was extracted separately from 18 unrelated individual trees of *E. grandis* and 18 of *E. globulus*. For each species three bulks of six individuals were prepared with equimolar amounts of picogreen quantified DNA. DNA samples were delivered to Floragenex who carried out the RAD reduced representation library preparation using PstI and Illumina 75 bp single-end sequencing on a GAIIx. Raw sequence data was filtered for quality and mapped onto the 11 chromosomes of the *E. grandis* reference genome available in Phytozome. SNPs in the short sequence tags were called for nucleotides with quality Q> 30 at the position and a minimum of 6X coverage.

## Results and conclusions

**Figure 1:**
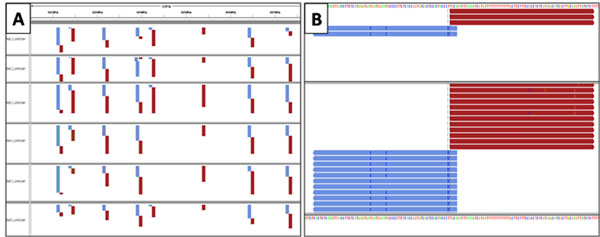
Screenshots of RAD sequencing tags mapped onto the Eucalyptus genome. (A) RAD tags generated from the six bulked samples (top to bottom) from 8 sequential PstI sites showing the variability in the presence or absence of tags across samples and in different directions (red = forward; blue=reverse); (B) close-up screenshot of SNPs detected in the RAD tags of two bulked samples.

The average sequencing depth exceeded 28X for all bulked samples, providing a minimum estimated ~5X coverage for each individual present in each bulk providing a 93.75% probability of detecting a heterozygous SNP position. With 18 individuals per species (36 chromosomes), the probability of detecting a SNP allele with a Minimum Allele Frequency (MAF) > 0.1 is > 95% [5] therefore providing good power to select informative SNPs in each species separately and even more so in both species simultaneously. RAD sequence tags may be present or absent in specific individuals depending on the presence of the PstI restriction site providing large numbers of dominant markers; SNPs detected within the aligned tags provide additional co-dominant markers (Figure 1).

Out of a total of 200,712 SNPs declared with high confidence, 42,300 were simultaneously polymorphic in the two species while the remaining were fixed in one or the other. These 42,300 SNPs provide an average density of one SNP every 14 kbp in the Eucalyptus genome. These SNPs could be immediately used to select an evenly spaced set of SNPs for the development of a high density SNP genotyping platform.

**Figure 2:**
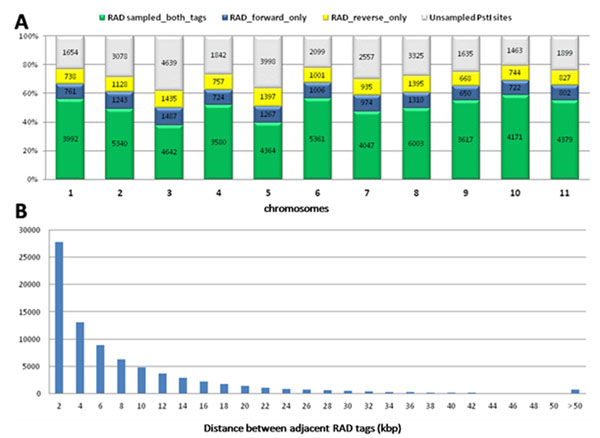
(A) Sampling of the PstI sites in the 11 chromosomes of *Eucalyptus* provided by RAD sequencing. Counts (into bars) and percent (Y axis) of unsampled PstI sites, RAD sequence tags generated in both directions out of the PstI site or in single directions (fwd or rev); (B) Distribution of the distances between adjacent RAD sequencing tags across the 605 Mbp of the *Eucalyptus* genome.

Taken together, the RAD tags plus the SNPs into them provide excellent marker density for applications such as Genomic Selection [6]. Besides the RAD method, Elshire et al. [7] recently described a straightforward method of genotyping-by-sequencing. Additionally the DArT complexity reduction protocol has also been streamlined based on NGS for a number of plant genomes including *Eucalyptus* (see Sansaloni et al. this meeting). All these NGS based genotyping methods will cause a paradigm shift in our ability to carry out high density, high throughput and low-cost genotyping of large numbers of samples, unlocking incredible opportunities in forest tree genetics and breeding in the years to come.
